# Neu5Gc-mediated high-affinity interaction is dispensable for CD22 cis-ligands to regulate B cell signaling

**DOI:** 10.1016/j.jbc.2024.107630

**Published:** 2024-08-03

**Authors:** Chizuru Akatsu, Yuko Naito-Matsui, Hajjaj H.M. Abdu-Allah, Akihiro Imamura, Wang Long, Hideharu Ishida, Hiromu Takematsu, Takeshi Tsubata

**Affiliations:** 1 Department of Immunology, Medical Research Institute, Tokyo Medical and Dental University, Tokyo, Japan; 2 Department of Molecular Cell Biology, School of Medical Sciences, Fujita Health University, Toyoake, Aichi, Japan; 3 Department of Applied Bio-organic Chemistry, Gifu University, Gifu, Japan; 4 Institute for Glyco-core Research (iGCORE), Gifu University, Gifu, Japan; 5 Department of Pathology, Nihon University School of Dentistry, Tokyo, Japan

**Keywords:** sialic acid, Neu5Gc, Siglec, CD22, Siglec-2, cis-ligand, high affinity ligand, B cell, BCR, signaling

## Abstract

CD22 (also known as Siglec-2) is an inhibitory receptor expressed in B cells. CD22 specifically recognizes α2,6 sialic acid and interacts with α2,6 sialylated membrane proteins expressed on the same cell (cis-ligands) and those derived from outside of the cell (trans-ligands). Previously, CD22 cis-ligands were shown to regulate the activity of CD22, thereby regulating both BCR ligation-induced signaling and low-level “tonic” signaling in the absence of BCR ligation that regulates the survival and differentiation of B cells. Mouse CD22 prefers Neu5Gc to Neu5Ac thereby binding to α2,6-linked Neu5Gc with high affinity. Although human CD22 binds to a distinct α2,6 sialylated glycan with high affinity, expression of high-affinity ligands is regulated in a conserved and stringent manner. However, how high- *versus* low-affinity CD22 ligands regulate B cells is poorly understood. Here we demonstrate that the interaction of CD22 with the endogenous ligands enhances BCR ligation-induced signaling but reduces tonic signaling in *Cmah*^−/−^ mouse B cells deficient in Neu5Gc as well as wild-type B cells. Moreover, *Cmah*^−/−^ B cells do not show alterations in the phenotypes correlated to tonic signaling. These results indicate that low-affinity interaction of the CD22 cis-ligands with CD22 is sufficient for the regulation of B cell signaling, and suggest that expression of high-affinity CD22 ligands might be involved in the regulation of B cells by competing for the binding of CD22 with exogenous trans-ligands of CD22.

CD22 (also known as Siglec-2) is a member of the sialic acid-binding immunoglobulin-like lectin (Siglec) family preferentially expressed in B lymphocytes (B cells) (1, 2). CD22 contains four immunoreceptor tyrosine-based inhibition motifs (ITIMs) in the cytoplasmic region (3). Upon phosphorylation, these ITIMs recruit and activate SH2-containing protein tyrosine phosphatase 1 (SHP-1), thereby negatively regulating signaling through the B cell antigen receptor (BCR) (1, 2). The extracellular region of CD22 contains the N-terminal lectin domain that specifically recognizes α2,6 sialic acid (4), and interacts with α2,6 sialylated molecules as ligands. Mouse CD22 prefers Neu5Gc to Neu5Ac in recognition of α2,6 sialic acid (5). Consequently, CD22 binds to Neu5Gcα2-6Galβ1-4GlcNAc with high affinity. Human cells do not produce Neu5Gc because of a loss-of-function mutation in the gene encoding cytidine monophosphate (CMP)-N-acetylneuraminic acid hydroxylase (Cmah) that converts CMP-Neu5A to CMP-Neu5Gc (6). Instead, human CD22 binds to the sulfated glycan Neu5Acα2-6Galβ1-4(6-sulfo)GlcNAc with high affinity (7). Expression of high-affinity CD22 ligands in both B and T cells is highly regulated. When mouse B and T cells are activated *in vitro*, expression of Cmah is markedly reduced, resulting in a reduction in the high-affinity CD22 ligands (8, 9). Upon immunization, B cells activated by antigen stimulation undergo clonal expansion in the structure called germinal centers where B cells undergo affinity maturation of antibodies through somatic hypermutation of Ig V genes and differentiation to memory B cells and long-lived plasma cells. Although the high-affinity ligands for human and mouse CD22 are structurally different, expression of the high-affinity CD22 ligands are markedly reduced in germinal center B cells (7, 8).

CD22 constitutively interacts with α2,6 sialylated molecules expressed on the same cell (cis-ligands) but can interact with those expressed on other cells (trans-ligands) (10). CD22 interacts with other CD22 molecules as a major ligand, thereby forming the CD22 cluster on the B cell surface (11). CD22 also interacts with other α2,6 sialylated molecules such as IgM and CD45 (12). Several lines of evidence suggest that the interaction of CD22 with these ligands regulates signaling and phenotypes of B cells (2, 13, 14, 15, 16, 17). Roles of interaction between CD22 and α2,6 sialylated ligands have been addressed using ST6Gal1^−/−^ B cells that lack α2,6 sialic acid (13, 15, 16). However, the roles of ligand interaction cannot be determined by the finding in *S*t*6*g*al1*^−/−^ B cells alone because α2,6 sialic acid binds to the other molecules such as Siglec-G as well as CD22 (18). In contrast, the synthetic sialoside GSC718 that we generated previously inhibits ligand binding of CD22 by binding to CD22 with 10,000-fold higher affinity than α2,6 sialylated ligands (19). Importantly, GSC718 does not inhibit sialylated ligand binding of other mouse Siglecs such as Siglec-1, CD33, MAG, Siglec-E, Siglec-G and Siglec-H (16). These results indicate that GSC718 specifically inhibits ligand binding of CD22. The specificity of GSC718 to CD22 is probably generated by the binding of GSC718 to the region of CD22 that is not conserved with other Siglecs.

Previous studies show that BCR ligation-induced signaling is reduced in *S*t*6*g*al1*^−/−^ B cells but not in *S*t*6*g*al1*^−/−^ *Cd22*^−/−^ B cells (13, 15, 16). Although α2,6 sialic acid binds to multiple molecules including CD22, the requirement of CD22 in the reduction of BCR ligation-induced signaling strongly suggests that loss of ligand-CD22 interaction is responsible for reduced BCR ligation-induced signaling in *S*t*6gal1*^−/−^ B cells. This notion is supported by the finding that BCR ligation-induced signaling is reduced by treatment with GSC718 which specifically inhibits ligand binding of CD22 (16). GSC718 no longer alters BCR ligation-induced signaling in *Cd22*^−/−^ or *S*t*6*g*al1*^−/−^ B cells, further supporting the specificity of GSC718 to CD22. These results clearly indicate that ligand-CD22 interaction augments BCR ligation-induced signaling probably by inhibiting CD22-mediated signal inhibition.

*Cd22*^−/−^ B cells show phenotypic alterations such as a reduction in the number of marginal zone (MZ) B cells (16, 20) and a reduction in the surface IgM level in follicular (FO) B cells (16, 21, 22, 23, 24). These phenotypic alterations are opposite to the changes in B cells deficient in the signaling molecules essential for BCR signaling such as Btk and PLCγ2 (25, 26, 27). It is already established that BCR transmits low-level signaling called tonic signaling in the absence of antigen stimulation (28, 29). Therefore, the phenotypic changes in BCR signaling-deficient B cells appear to be caused by a deficiency in tonic signaling, and the inverse phenotypic changes in *Cd22*^−/−^ B cells suggest that CD22 down-modulates tonic signaling as well as BCR ligation-induced signaling.

Previously, we and other groups demonstrated that *S*t*6*g*al1*^−/−^ B cells show phenotypic changes similar to *Cd22*^−/−^ B cells (16, 20), indicating that α2,6 sialic acid as well as CD22 down-regulates tonic signaling in B cells. Although Siglec-G is an inhibitory receptor expressed in B cells and interacts with α2,6 sialic acid (18), *Siglec*g^−/−^ B cells do not show alteration in BCR ligation-induced signaling or phenotypes in conventional B cells (30), indicating that Siglec-G is dispensable for the regulation of both BCR ligation-induced and tonic signaling in conventional B cells. Therefore, α2,6 sialic acid regulates tonic signaling in conventional B cells probably by regulating CD22 but not Siglec-G. Together, these findings suggest that the interaction of CD22 with the α2,6 sialylated ligands regulates both tonic and BCR ligation-induced signaling. However, it is not yet clear whether these regulations require high-affinity interaction of CD22 with the ligands. Here we analyze both BCR ligation-induced and tonic signaling in *Cmah*^−/−^ B cells, and show that low-affinity interaction of CD22 with the ligands is sufficient for functional regulation of CD22 by the ligands.

## Results

### Neu5Gc is dispensable for the regulation of CD22 by endogenous ligands in BCR-ligated B cells

**Figure 1:**
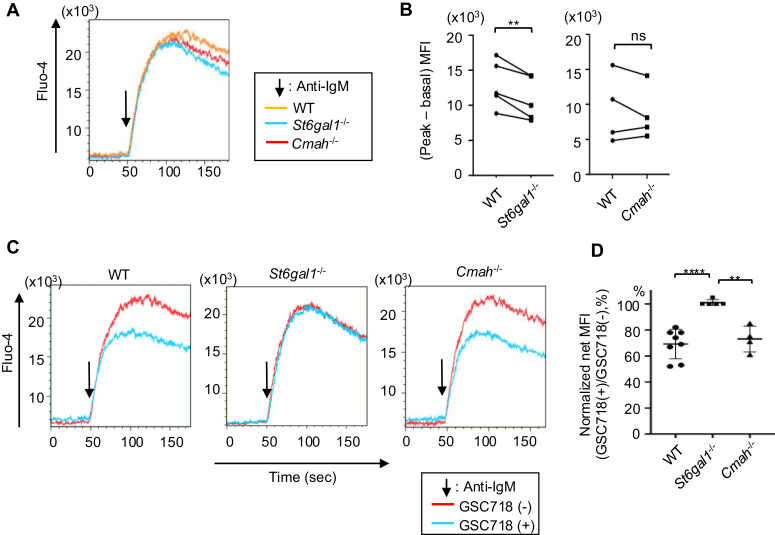
**Neu5Gc is dispensable for upregulation of BCR ligation-induced signaling by ligand-CD22 interaction.** Spleen B cells were obtained from wild type (WT), *St6gal1*^−/−^ and *Cmah*^−/−^ C57BL/6 mice. Cells were loaded with Fluo-4, and stimulated with anti-IgM antibody without (*A–D*) or with (*C* and *D*) GSC718. Intracellular Ca^2+^ level was measured by flow cytometry. Representative data are shown (*A* and *C*). The *arrows* indicate the time point when the stimulants were added. The basal MFI was subtracted from the peak MFI ((peak - basal) MFI) (*B* and *D*). Normalized net MFI (GSC718(+)/GSC718(−)) was calculated by the following formula. $\text{Normalized}\;\text{net}\;\text{MFI}\;\left(\text{GSC}718\left(+\right)/\text{GSC}718\left(-\right),\%\right)=\frac{\left(\text{peak}-\text{basal}\right)\text{MFI}\;\text{with}\;\text{GSC}718}{\left(\text{peak}-\text{basal}\right)\text{MFI}\;\text{without}\;\text{GSC}718}\;\times \;100$. Data were analyzed by paired Student’s *t* test (n = 5 for *St6gal1*^*−*/−^ and n = 4 for *Cmah*^−/−^) (*B*) or one-way ANOVA followed by Tukey’s multiple comparison test (n = 8 for WT, n = 5 for *St6gal1*^*−*/−^, n = 4 for *Cmah*^−/−^) (*D*). ns *p* > 0.05, ∗∗*p* < 0.01, ∗∗∗∗*p* < 0.001.

To address the role of high-affinity CD22 ligands in the regulation of BCR signaling, we examined BCR ligation-induced Ca^2+^ signaling in wild-type, *St6gal1*^*−*/−^ and *Cmah*^−/−^ B cells in the presence or absence of GSC718. *St6gal1*^−/−^ B cells show a significant reduction in BCR ligation-induced signaling compared to wild-type B cells (Fig. 1, *A* and *B*), indicating that α2,6 sialic acid enhances BCR signaling. Previously, we showed that the synthetic sialoside GSC718 specifically inhibits ligand-CD22 interaction (16). Treatment with GSC718 reduces BCR ligation-induced Ca^2+^ flux in wild-type B cells but not *St6gal1*^−/−^ B cells (Fig. 1, *C* and *D*), indicating that ligand-CD22 interaction enhances BCR signaling. These results are consistent with the previous findings (13, 15, 16), and suggest that α2,6 sialic acid enhances BCR signaling by mediating ligand-CD22 interaction. Both ST6Gal1 deficiency and GSC718 disrupt the binding of CD22 with endogenous ligands including both cis- and trans-ligands. Therefore, these results suggest that BCR ligation-induced signaling is augmented by the interaction of CD22 with endogenous ligands. BCR ligation-induced signaling in *Cmah*^−/−^ B cells is comparable to that in wild-type B cells (Fig. 1, *A* and *B*), and treatment with GSC718 reduces BCR ligation-induced signaling in *Cmah*^−/−^ B cells as well as wild-type B cells (Fig. 1, *C* and *D*). This result indicates that Neu5Gc required for high-affinity interaction of α2,6 sialylated ligands to CD22 is dispensable for the regulation of BCR ligation-induced signaling by ligand-CD22 interaction, and suggests that high-affinity interaction of CD22 with the endogenous ligands is dispensable for the regulation of the CD22 activity by endogenous ligands of CD22 in BCR-ligated B cells.

### *Cmah*^−/−^ B cells do not show phenotypes correlated with altered tonic signaling

**Figure 2:**
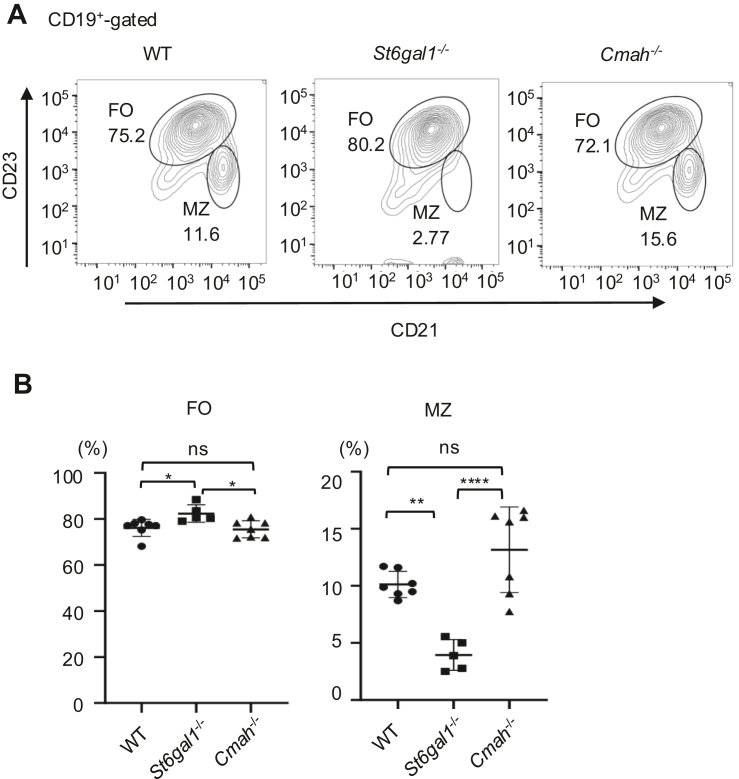
**Fraction of MZ B cells is reduced in *S******t******6******g******al1***^**−/−**^**but not *Cmah***^***−*/−**^**B cells.** Spleen cells were obtained from wild-type (WT), *St6gGal1*^*−*/−^ and *Cmah*^−/−^ C57BL/6 mice. CD19^+^ or B220^+^ cells were analyzed for CD21 and CD23 by flow cytometry. Representative data are shown (*A*). Percentages of CD21^lo^CD23^hi^ FO B cells and CD21^hi^ CD23^lo^ MZ B cells are indicated. Percentages of FO and MZ B cells and Mean ± SD are shown (n = 7 for WT, n = 5 for *St6gal1*^−/−^ and n = 7 for *Cmah*^−/−^) (*B*). Data were analyzed by one-way ANOVA followed by Tukey’s multiple comparison test. ns *p* > 0.05, ∗*p* < 0.05, ∗∗*p* < 0.01, ∗∗∗∗*p* < 0.001. MZ, marginal zone.

**Figure 3:**
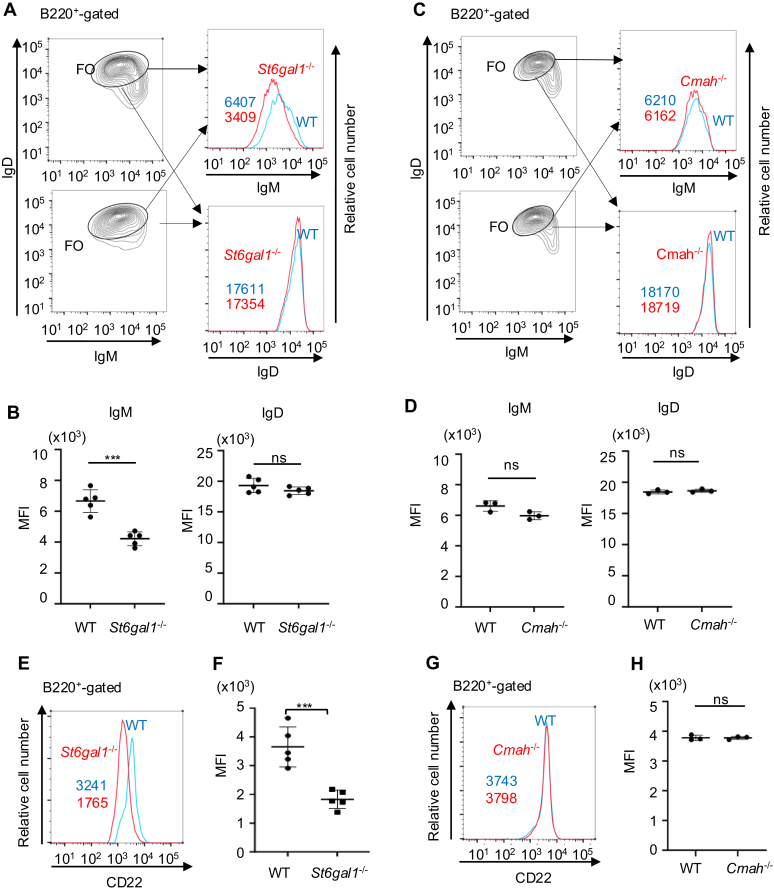
**Surface expression of IgM and CD22 is reduced in *S******t******6******g******al1***^**−/−**^**but not *Cmah***^**−/−**^**B cells.** Spleen cells were obtained from wild-type (WT), *St6gal1*^−/−^ and *Cmah*^−/−^ C57BL/6 mice. *A–D*, B220^+^IgM^lo^IgD^hi^ FO B cells from WT (*A–D*), *St6gal1*^−/−^ (*A* and *B*) and *Cmah*^−/−^ mice (*C* and *D*) were gated and analyzed for IgM and IgD expression. Representative data are shown (*A* and *C*). MFIs are indicated. MFIs of IgM and IgD (n = 3) and Mean ± SD are shown (*B* and *D*). *E-H*, B220^+^ cells from WT (*E–H*), *St6gal1*^*−*/−^ (*E* and *F*) and *Cmah*^−/−^ (*G* and *H*) mice were gated and analyzed for CD22. Representative data are shown (*E* and *G*). MFIs of CD22 are indicated. MFIs of CD22 (n = 3) and Mean ± SD are shown (*F* and *H*). Data were analyzed by Student’s *t* test. ns: not significant, ∗∗∗*p* < 0.005.

To address whether Cmah regulates B cell phenotypes correlated with the tonic signaling level, we analyzed the fractions of CD19^+^CD21^hi^CD23^lo^ MZ B cells and CD19^+^CD21^lo^CD23^hi^ FO B cells in the spleen, and the surface IgM level in FO B cells in Cmah^−/−^ and *St6gal1*^−/−^ mice. *St6gal1*^−/−^ mice show a reduction in the percentage of MZ B cells in the spleen (Fig. 2), and a reduction in the surface IgM but not IgD level (Fig. 3, *A* and *B*) compared to wild-type mice. These results are consistent with the previous study (16) and suggest that α2,6 sialic acid reduces the tonic signaling level in B cells. In contrast, both the percentage of MZ B cells in the spleen (Fig. 2) and the surface IgM level in FO B cells (Fig. 3, *C* and *D*) are not significantly altered in *Cmah*^−/−^ mice compared to those in wild-type mice although the sample numbers are relatively small. These results suggest that Cmah deficiency does not alter tonic signaling in B cells. Taken together, α2,6 sialic acid down-modulates tonic signaling probably by mediating the interaction of CD22 with the endogenous ligands, but Neu5Gc required for high-affinity interaction of CD22 with the ligands is dispensable in the regulation of tonic signaling, suggesting that regulation of tonic signaling by ligand-CD22 interaction does not require high-affinity interaction of CD22 with the ligands.

The surface expression of CD22 was shown to be reduced in *St6gal1*^−/−^ B cells (13, 16). This result suggests that ligand-CD22 interaction up-regulates surface expression of CD22, although the mechanism is not yet known. Whereas *St6gal1*^−/−^ B cells show a significant reduction in the surface CD22 level compared to wild-type B cells (Fig. 3, *E* and *F*), the surface CD22 level in *Cmah*^−/−^ B cells is comparable to that in wild-type B cells (Fig. 3, *A* and *H*), suggesting that high-affinity interaction of CD22 with endogenous ligands is not required for up-regulation of the surface CD22 level.

### Restoration of BCR signaling in immunodeficient *Cd45*^−/−^ B cells by CD22 ligands does not require Neu5Gc with CD22

**Figure 4:**
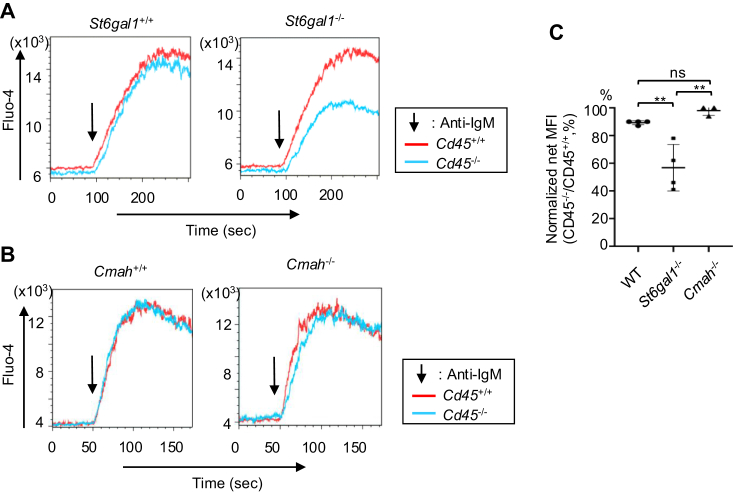
**α2,6 sialic acid but not Neu5Gc is required for CD22 ligand-mediated restoration of BCR ligation-induced signaling in *C******d******45***^***−*/−**^**B cells.** BCR ligation-induced Ca^2+^ signaling in spleen B cells from wild-type (WT) C57BL/6 mice (*A*–*C*) or C57BL/6 mice deficient in either ST6Gal1 (*A* and *C*), Cmah (*B* and *C*), CD45 (*A–C*), both CD45 and ST6Gal1 (*A* and *C*) or both CD45 and Cmah (*B* and *C*). Cells were loaded with Fluo-4, and stimulated with anti-IgM antibody. Intracellular Ca^2+^ level was measured by flow cytometry. Representative data are shown (*A* and *B*) The *arrows* indicate the time point when anti-IgM was added. The basal MFI was subtracted from the peak MFI ((peak - basal) MFI) (*C*). Normalized net MFI (*Cd45*^−/−^/*Cd45*^+/+^) was calculated by the following formula. $\text{Normalized}\;\text{net}\;\text{MFI}\;\left({\mathrm{Cd}45}^{-/-}/{\mathrm{Cd}45}^{+/+},\%\right)=\frac{\left(\text{peak}-\text{basal}\right)\text{MFI}\;\text{in}\;{\mathrm{Cd}45}^{-/-}\;B\;\text{cells}\;}{\left(\text{peak}-\text{basal}\right)\text{MFI}\;\text{in}\;{\mathrm{Cd}45}^{+/+}\;B\;\text{cells}\;}\;\times \;100$. Data were analyzed by one-way ANOVA followed by Tukey’s multiple comparison test (n = 4 for WT and *St6gal1*^−/−^, n = 3 for *Cmah*^−/−^). ns *p* > 0.05, ∗∗*p* < 0.01.

CD45 is a receptor-type tyrosine phosphatase required for activation of Src-family kinases (31). Although Src-family kinases are essential for BCR signaling (32, 33), *Cd45*^−/−^ B cells show almost normal BCR ligation-induced signaling (16), suggesting that the signaling defect caused by CD45 deficiency is restored. This restoration of BCR signaling requires both CD22 and α2,6 sialic acid because both *Cd45*^−/−^*St6gal1*^−/−^ and *Cd45*^−/−^*Cd22*^−/−^B cells show markedly reduced BCR ligation-induced signaling compared to *St6gal1*^−/−^ and *Cd22*^−/−^ B cells, respectively. Therefore, ligand-CD22 interaction appears to be involved in the restoration of BCR ligation-induced signaling in *Cd45*^−/−^ B cells. To address whether high-affinity CD22 ligands are required for the restoration of BCR ligation-induced signaling in *Cd45*^−/−^ B cells, we examined BCR signaling in *Cmah*^−/−^*Cd45*^−/−^ B cells. Ca^2+^ flux induced by treatment with anti-IgM in *Cd45*^−/−^ B cells is comparable to that in *Cd45*^+/+^ B cells (Fig. 4, *A* and *B* left panels) as described previously (16, 34, 35). *St6gal1*^−/−^ *Cd45*^−/−^ B cells show markedly reduced BCR ligation-induced Ca^2+^ signaling compared to *St6gal1*^−/−^ *Cd45*^+/+^ B cells (Fig. 4*A* right panel), consistent with the previous study (16). In contrast, *Cmah*^−/−^*Cd45*^−/−^ B cells show anti-IgM-induced Ca^2+^ signaling comparable to that in *Cmah*^−/−^ B cells (Fig. 4*B* right panel). This result indicates that BCR ligation-induced signaling is restored in *Cmah*^−/−^*Cd45*^−/−^ B cells as well as *Cmah*^+/+^*Cd45*^−/−^ B cells, and suggests that high-affinity interaction of CD22 with the ligands is dispensable for restoration of BCR ligation-induced signaling in signaling-deficient *Cd4*5^−/−^ B cells.

### Cmah does not regulate basal signaling in B cells

**Figure 5:**
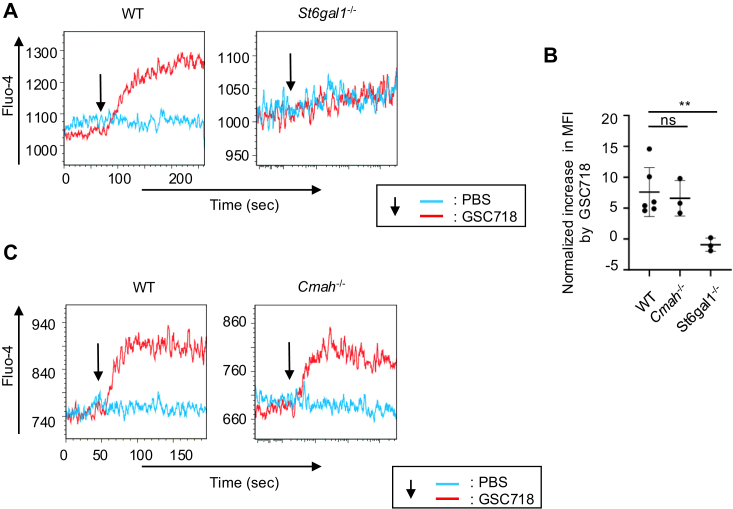
**Neu5Gc is dispensable for down-regulation of basal Ca**^**2+**^**signaling in B cells by ligand-CD22 interaction.** Spleen B cells were obtained from wild type (WT), *St6gal1*^−/−^ and *Cmah*^−/−^ C57BL/6 mice. Cells were loaded with Fluo-4, and stimulated with or without GSC718. Intracellular Ca^2+^ level was measured by flow cytometry. Representative data are shown (*A* and *C*). The *arrows* indicate the time point when GSC718 was added. Normalized increase in MFI by GSC718 was calculated by the following formula (*B*) to correct the difference in the basal MFI between experiments. $\text{Normalized}\;\text{increase}\;\text{in}\;\text{MFI}\;\text{by}\;\text{GSC}718\;\left(\%\right)=\frac{\;\text{highest}\;\text{MFI}\;\text{with}\;\text{GSC}718-\text{highest}\;\text{MFI}\;\text{without}\;\text{GSC}718}{\text{highest}\;\text{MFI}\;\text{without}\;\text{GSC}718}\;\times \;100$. Data were analyzed by one-way ANOVA followed by Dunnet’s multiple comparison test (n = 4). ns *p* > 0.05, ∗∗*p* < 0.01.

Previously, we demonstrated that GSC718 increases the basal Ca^2+^ level that appears to correlate to the tonic signaling level, and this response requires both CD22 and ST6Gal1 (16). This result suggests that ligand-CD22 interaction down-regulates the basal Ca^2+^ signaling. To address whether high-affinity ligand interaction of CD22 is required for this regulation, we treated wild-type, *St6gal1*^−/−^ and *Cmah*^−/−^ B cells with GSC718 and analyzed the basal Ca^2+^ level. As shown previously, GSC718 increases the Ca^2+^ level in wild-type but not *St6gal1*^−/−^ B cells (Fig. 5, *A* and *B*). In contrast, GSC718 increases the Ca^2+^ level in *Cmah*^−/−^ as well as wild-type B cells (Fig. 5, *B* and *C*). This result clearly demonstrates that high-affinity ligand interaction of CD22 is dispensable for the regulation of basal Ca^2+^ signaling.

## Discussion

In this study, we showed reduced BCR ligation-induced signaling and B cell phenotypes correlated to tonic signaling in *St6gal1*^−/−^ but not *Cmah*^−/−^ B cells. This result indicates that B cell signaling and phenotypes are regulated by α2,6 sialic acid that mediates ligand binding of CD22 (4) whereas Neu5Gc required for high-affinity ligand interaction of CD22 (5) does not regulate B cell signaling or phenotypes. Although α2,6 sialic acid is recognized by the other molecules including Siglec-G (18) as well as CD22, reduction in BCR ligation-induced signaling in *St6gal1*^−/−^ B cells requires CD22 (15), indicating that ligand-CD22 interaction is responsible for the regulation of BCR ligation-induced signaling by α2,6 sialic acid. *Siglecg*^−/−^ B cells do not show alterations in BCR signaling and B cell phenotypes (30), and *Cd22*^−/−^ B cells show similar phenotypic alterations to *St6gal1*^−/−^ B cells (21, 23, 24). Therefore, CD22 appears to mediate the regulation of B cells by α2,6 sialic acid although analysis of *St6gal1*^−/−^ B cells *per se* does not exclude the involvement of other molecules that recognize α2,6 sialic acid. Normal BCR ligation-induced signaling and B cell phenotypes correlated to tonic signaling in *Cmah*^−/−^ B cells suggest that ligand-CD22 interaction does not require Neu5Gc-containing high-affinity ligands for the regulation of BCR ligation-induced and tonic signaling. These conclusions on the roles of ligand-CD22 interaction in B cell signaling are further supported by the results using GSC718 which specifically inhibits ligand binding of CD22 (16). Here we show that treatment with GSC718 reduces BCR ligation-induced Ca^2+^ signaling but increases basal Ca^2+^ signaling in *Cmah*^−/−^ B cells as well as wild-type B cells. In contrast, GSC718 does not modulate BCR ligation-induced or basal Ca^2+^ signaling in *Cd22*^−/−^ or *St6gal1*^−/−^ B cells (16), suggesting that GSC718 regulates B cell signaling by specifically inhibiting ligand-CD22 interaction. Therefore, Neu5Gc-containing high-affinity CD22 ligands are not required for the regulation of BCR ligation-induced and tonic signaling by ligand-CD22 interaction.

We further showed that the regulation of B cell signaling by ligand-CD22 interaction rescues BCR signaling in *Cd45*^−/−^ B cells deficient in activation of Src-family kinases probably by deleting B cells with the low tonic signaling level during B cell development (16). This finding suggests that ligand-CD22 interaction is involved in the selective development of BCR signaling-competent B cells. Here we show that BCR signaling is restored in *Cd45*^−/−^*Cmah*^−/−^ B cells as well as *Cd45*^−/−^ B cells, indicating that Neu5Gc is not required for the selective development of BCR signaling-competent B cells probably because Neu5Gc is dispensable for the regulation of tonic signaling.

Previously, we showed that the interaction of other CD22 molecules and BCR with CD22 up-regulates BCR ligation-induced and down-regulates tonic signaling, respectively (36). Because these regulations involve interaction between the cytoplasmic regions of both CD22 and the ligands, cis-interaction of CD22 with these ligands is essential (2). Here we show that maintenance of the surface CD22 level requires ST6Gal1 but not Cmah, although the mechanism is not yet clear. Previously, cis-interaction of CD22 with β7 integrin was shown to be required for maintenance of surface β7 expression in B cells by inhibiting endocytosis of β7 (37). Therefore, cis-interaction of CD22 with another CD22 may be involved in the maintenance of surface CD22 by inhibiting its endocytosis. Taken together, our results on Cmah^−/−^ B cells suggest that Neu5Gc is not required for the regulation of BCR signaling and CD22 expression by the interaction of CD22 with cis-ligands. Therefore, regulation of CD22 by cis-ligands may not require a high affinity interaction of CD22 with the ligands.

Expression of high-affinity CD22 ligands is tightly regulated in a conserved manner between human and mouse although the structure of α2,6 sialic acid that binds to CD22 with high affinity is different between humans and mice (7, 8, 9), suggesting a crucial role of high-affinity CD22 ligands. In mice, expression of the high-affinity ligands depends on the expression of Cmah, and Cmah expression is markedly reduced upon B cell activation (8, 9). Expression of Cmah is almost completely lost in both B and T cells in germinal centers. Recently, Macauley and his colleagues showed that differentiation of plasma cells from germinal center B cells is defective in Cmah knock-in mice, in which Cmah expression is not reduced in germinal center B cells (38), suggesting that Neu5Gc plays a role in B cell responses to antigens. Here we show that Neu5Gc does not play a role in the regulation of B cell signaling by endogenous CD22 ligands. In contrast, the interaction of CD22 with exogenous sialylated molecules is augmented in *Cmah*^−/−^ B cells (8, 39) probably because binding of trans-ligands to CD22 is not well competed by low-affinity cis-ligands expressed in *Cmah*^−/−^ B cells. Therefore, Neu5Gc may play a role in the regulation of B cell responses by suppressing the interaction of CD22 with exogenous CD22 ligands, or by interacting molecules other than CD22.

## Experimental procedures

### Mice

C57BL/6 mice were purchased from Sankyo Labo Service or CLEA Japan. *Cd45*^−/−^ mice (40) (a gift from Dr H. Kishi at Toyama University), *Cmah*^−/−^ mice (8) and *St6gal1*^−/−^ mice (13) (a gift from Dr J. D. Marth at the University of California Santa Barbara) on C57BL/6 background were bred and maintained in the animal facility of Tokyo Medical and Dental University or Fujita Health University under specific pathogen-free conditions. Mice were used at 8 to 15 weeks old. Experiments were approved by the Institutional Animal Care and Use Committee of Tokyo Medical and Dental University or Fujita Health University and were performed according to our institutional guidelines.

### Cells and reagents

Mouse spleen B cells were prepared as described previously (41). Synthetic sialoside GSC718 was synthesized as described previously (19).

### Flow cytometry

Mouse spleen cells were treated with anti-FcγRII/III antibody 2.4G2 for 15 min to block FcγRII/III-mediated nonspecific binding, and stained with the following antibodies: Anti-mouse CD22 (F239) (12) and anti-mouse IgM (115-006-020, Jackson ImmunoResearch) conjugated with Alexa647 and Pacific Blue, respectively, using Alexa Fluor 647 and Pacific Blue antibody conjugation kits (Invitrogen), FITC-conjugated anti-mouse CD21 (7E9, BioLegend; 7G6, BD PharMingen), PE-conjugated anti-mouse CD23 (B3B4, eBioscience), biotin-conjugated anti-mouse CD23 (B3B4, BD PharMingen), R-PE-conjugated streptavidin (Invitrogen), biotin-conjugated anti-mouse CD19 (eBio13, eBioscience), Pacific Blue-conjugated streptavidin (Invitrogen), APC-conjugated anti-mouse CD45R/B220 (RA3-6B2, BD Pharmingen), FITC-conjugated anti-mouse CD45R/B220 (RA3-6B2, BD Pharmingen), Alexa647-conjugated anti-mouse IgD (11-26c, eBioscience), FITC-conjugated anti-mouse IgM (1020-02, SBA). Cells were analyzed using a FACSVerse (BD), a FACSCalibur (BD), or a FACSAria (BD). Data were analyzed by FlowJo (Treestar).

### Measurement of intracellular Ca^2+^ concentration

Mouse spleen B cells (2 × 10^6^) were incubated with 5 μg/ml Fluo-4 AM (Invitrogen) for 30 min. After washing, cells were stimulated with 10 μg/ml F(ab’)_2_ fragments of goat anti-mouse IgM (115-006-020, Jackson ImmunoResearch), 100 μM GSC718, or both. Fluo-4 fluorescence was measured continuously using a CyAn (Beckman Courter). Data were analyzed by FlowJo (Treestar).

### Statistical analysis

Data were analyzed by one-way ANOVA followed by Tukey’s or Dunnet’s multiple comparison test. Alternatively, data were analyzed by Student’s *t* test. All the statistical analysis was done using GraphPad Prism 5.0 software (GraphPad). *p* values less than 0.05 were regarded as statistically significant.

## Data availability

All data are contained in the manuscript.

## Conflict of interest

The authors declare that they have no conflicts of interest with the contents of this article.
